# Memory Function in Feeding Habit Transformation of Mandarin Fish (*Siniperca chuatsi*)

**DOI:** 10.3390/ijms19041254

**Published:** 2018-04-22

**Authors:** Yaqi Dou, Shan He, Xu-Fang Liang, Wenjing Cai, Jie Wang, Linjie Shi, Jiao Li

**Affiliations:** College of Fisheries, Chinese Perch Research Center, Huazhong Agricultural University, No.1, Shizishan Street, Hongshan District, Wuhan, Hubei Province, 430070, China; Freshwater Aquaculture Collaborative Innovation Center of Hubei Province, Key Lab of Freshwater Animal Breeding, Ministry of Agriculture, Wuhan 430070, China; douyaqi@outlook.com (Y.D.); wenjing_caicai@163.com (W.C.); scofield_wj@163.com (J.W.); shilinjie824@163.com (L.S.); lijiao0519@foxmail.com (J.L.)

**Keywords:** mandarin fish (*Siniperca chuatsi*), memory, feeding habit transformation, training, repeated training, DNA methylation

## Abstract

Mandarin fish refuse dead prey fish or artificial diets and can be trained to transform their inborn feeding habit. To investigate the effect of memory on feeding habit transformation, we compared the reaction time to dead prey fish and the success rate of feeding habit transformation to dead prey fish with training of mandarin fish in the 1st experimental group (trained once) and the 2nd experimental group (trained twice). The mandarin fish in the 2nd group had higher success rate of feeding habit transformation (100%) than those in the 1st group (67%), and shorter reaction time to dead prey fish (<1 s) than those in the 1st group (>1 s). Gene expression of cAMP responsive element binding protein I (*Creb I*), brain-derived neurotrophic factor (*Bdnf*), CCAAT enhancer binding protein delta (*C/EBPD*), fos-related antigen 2 (*Fra2*), and proto-oncogenes *c-fos* (*c-fos*) involved in long-term memory formation were significantly increased in the 2nd group after repeated training, and taste 1 receptor member 1 (*T1R1*), involved in feeding habit formation, was significantly increased in brains of the 2nd group after repeated training. DNA methylation levels at five candidate CpG (cytosine–guanine) sites contained in the predicted CpG island in the 5′-flanking region of *T1R1* were significantly decreased in brains of the 2nd group compared with that of the 1st group. These results indicated that the repeated training can improve the feeding habit transformation through the memory formation of accepting dead prey fish. DNA methylation of the *T1R1* might be a regulatory factor for feeding habit transformation from live prey fish to dead prey fish in mandarin fish.

## 1. Introduction

Animal feeding habits and feed-preferences not only affect growth characteristics and living habits, but also determine the aquaculture production costs and economic benefits. Although animal feeding habits and feed composition have been intensively investigated, little attention has been devoted to the transformation mechanism of inherent feeding habits of animals, especially in fish. Mandarin fish (*Siniperca chuatsi*), as a demersal piscivore, have a unique feeding habit. Once the fry of mandarin fish start feeding, they only accept live prey fish in the wild and refuse dead prey fish or artificial diets [1,2]. They can be trained to transform their innate feeding habit to accept dead prey fish [3].

Learning and memory could enable the organism to plastically respond to the changing environment. Increasing research has investigated the cognitive (learning) and memory characteristics of fish in the past few decades, including antipredator behavior [4,5,6,7,8], spatial cognition (orientation and migration) [9,10,11,12,13], learned recognition [14], social learning [4,15,16,17], mate choice [18,19,20], eavesdropping [4], and foraging activity [21,22]. Warburton [21] indicated that learning and memory system can play an important role in the foraging activity of fish. However, we still do not know whether learning and memory can play an important role on feeding habit transformation.

Feeding habit is a congenital behavior characteristic, which might be attributed to the integration of natural inheritance and acquired learning and memory [23,24]. Feeding habits of fish are regulated by a variety of factors, including (1) intrinsic genetic and physiological factors such as appetite and digestive tract structure and (2) external factors such as protein sources, food properties, and feed palatability [25]. Therefore, environmental changes might transform the feeding habit of fish. However, very little is currently known about the epigenetic regulation on learning and memory during feeding habit transformation. In mammals, epigenetic regulation has been reported to be involved in associational fear conditioning [26], extinction of learning fear [27], and spatial memory [28,29]. It has been reported that the experience-dependent epigenetic regulation is involved in long-term memory formation by the regulation of gene transcription [30].

The transcriptome sequencing of food preference in hybrid F_1_ of *Siniperca chuatsi* (♀) × *Siniperca scherzeri* (♂) mandarin fish was analyzed; 1986 and 4526 differentially expressed genes in feeders and nonfeeders (dead prey fish) were identified, respectively. The mRNA levels of proto-oncogenes *c-fos* (*c-fos*), fos-related antigen 2 (*Fra2*), immediate early gene *zif268* (*zif268*), cAMP responsive element binding protein I (*Creb I*), CCAAT enhancer binding protein delta (*C/EBPD*), brain-derived neurotrophic factor (*Bdnf*), and synaptotagmin-IV (*SytIV*) were significantly decreased in feeders, which might result in significant deficiency in memory retention of their natural food preference [2].

Previous study in mice suggested that taste 1 receptor member 1/3 (T1R1/T1R3) heterodimer might be a sole receptor for umami taste [31,32]. Proteins in the T1R family are expressed not only in taste bud cells in gustatory tissues, but also in the brain, gut, pancreas, and other non-gustatory tissues of various mammalian species [33,34,35,36,37]. Fasting affects expression level of *T1R1* in the mouse hypothalamus [35]. The expression of T1Rs in brain leads to an interesting question about their role. Pseudogenization of T1Rs in giant pandas (*Ailuropoda melanoleuca*) was related to its dietary switch from carnivore to herbivore [38,39]. Nonsynonymous single polymorphisms (nsSNP) in the coding region of *T1R1* and *T1R3* in humans contributed to the explanation of the inability to taste monosodium glutamate in non-tasters [40,41]. In regulation with gene transcription initiation, DNA methylation of CpG (cytosine–guanine) islands in gene control regions plays a critical role in gene silencing or activation through chromatin remodeling [42]. Therefore, it is necessary to note that the functions of DNA methylation in *T1R1* related to feeding habit transformation have not yet been realized.

In the present study, to investigate the effect of memory on feeding habit transformation, we compared the reaction time to dead prey fish and the success rate of feeding habit transformation from live prey fish to dead prey fish of mandarin fish in the 1st experimental group (trained once) and the 2nd experimental group (trained twice). The behavioral parameters, expression of genes involved in learning and memory, and DNA methylation levels in the CpGs of the *T1R1* gene involved in feed identification were examined in mandarin fish. This study improves the understanding of molecular mechanisms of learning and memory and the epigenetic regulation during the unique feeding habit transformation in mandarin fish.

## 2. Results

### 2.1. Success Rate of Feeding Habit Transformation and Reaction Time to Dead Prey Fish

**Figure 1:**
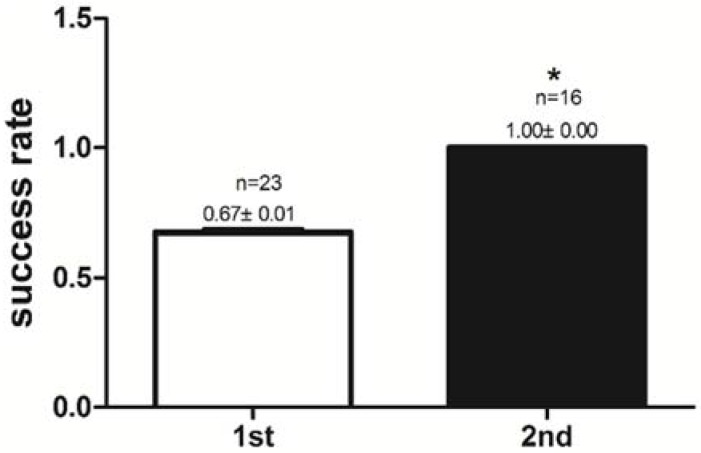
Comparison of the success rate of feeding habit transformation to dead prey fish of mandarin fish in the 1st experimental group (trained once) and the 2nd experimental group (trained twice). All values represent the mean ± standard error. * indicates significant differences (*P <* 0.05).

**Figure 2:**
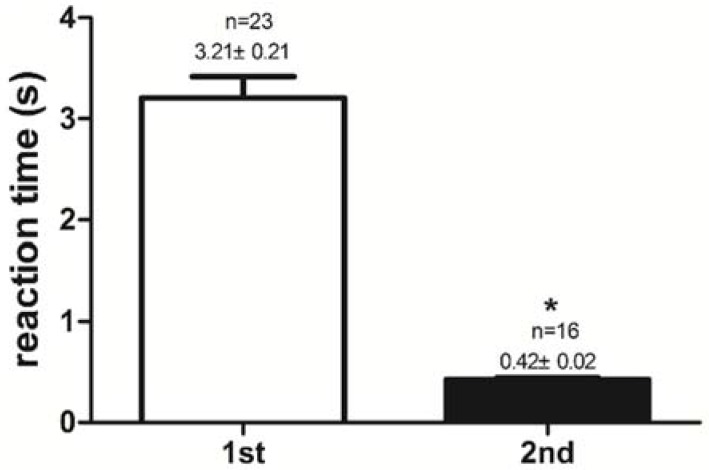
Reaction time(s) to dead prey fish of mandarin fish in the 1st experimental group (trained once) and the 2nd experimental group (trained twice). All values represent the mean ± standard error. * indicates significant differences (*P* < 0.05).

After pre-training for 6 days, all mandarin fish have great potentialities to accept dead prey fish. The success rates of feeding habit transformation to dead prey fish in the 1st experimental group (trained once) and the 2nd experimental group (trained twice) were compared. The success rate of feeding habit transformation to dead prey fish was 0.67 ± 0.01 (67%) in the 1st experimental group, and the success rate reached 1.00 ± 0.00 (100%) in the 2nd experimental group. The mandarin fish in the 2nd experimental group had a higher (*P <* 0.05) success rate than those in the 1st experimental group (Figure 1). Reaction time to dead prey fish of mandarin fish in the 1st and the 2nd experimental groups were 3.21 ± 0.21 s and 0.42 ± 0.02 s, respectively. The mandarin fish in the 2nd experimental group had shorter (*P <* 0.05) reaction times to dead prey fish (<1 s) than did those in the 1st experimental group (>1 s) (Figure 2). Once trained, mandarin fish preyed on dead prey fish faster.

### 2.2. Gene Expression Levels Analysis of Memory-Relative Genes in Mandarin Fish

**Figure 3:**
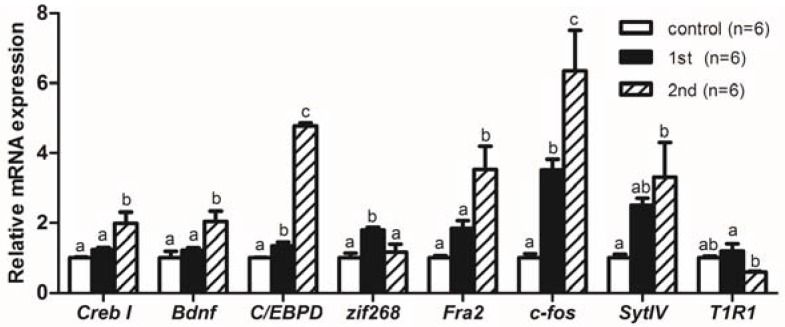
The cAMP responsive element binding protein I (*Creb I*), brain-derived neurotrophic factor (*Bdnf*), CCAAT enhancer binding protein delta (*C/EBPD*), immediate early gene *zif268* (*zif268*), fos-related antigen 2 (*Fra2*), proto-oncogenes *c-fos* (*c-fos*), synaptotagmin-IV (*SytIV*), and taste 1 receptor member 1 (*T1R1*) mRNA expression levels of mandarin fish in the control group, the 1st experimental group (trained once), and the 2nd experimental group (trained twice). The *C/EBPD*, *zif268*, and *c-fos* gene expression levels were significantly increased (*P* < 0.05) in the mandarin fish brains of the 1st experimental group compared with those of the control group. The *Creb I*, *Bdnf*, *C/EBPD*, *Fra2*, and *c-fos* gene expression levels were significantly increased (*P* < 0.05) and the *zif268* and *T1R1* gene expression levels were significantly reduced (*P* < 0.05) in mandarin fish brains of the 2nd experimental group compared with those of the 1st experimental group. The *SytIV* gene expression level was significantly increased (*P* < 0.05) in mandarin fish brains of the 2nd experimental group compared with that of the control group. All values represent the mean ± standard error, a–c indicate significant differences (*P* < 0.05).

As shown in Figure 3, after the first training, gene expression of *C/EBPD*, *zif268*, and *c-fos* were significantly increased (*P* < 0.05) in the mandarin fish brains of the 1st experimental group. Compared with the 1st experimental group, the expression levels of *Creb I*, *Bdnf*, *C/EBPD*, *Fra2*, and *c-fos* were significantly increased (*P* < 0.05) while the expression levels of *zif268* and *T1R1* were significantly reduced (*P* < 0.05) in the 2nd experimental group after repeated training. Compared with the control group, the gene expression of *SytIV* was significantly increased (*P* < 0.05) in the 2nd experimental group after two trainings.

### 2.3. DNA Methylation Analysis and Bisulphite Sequencing Polymerase Chain Reaction (BSP) of T1R1 Gene

**Figure 4:**
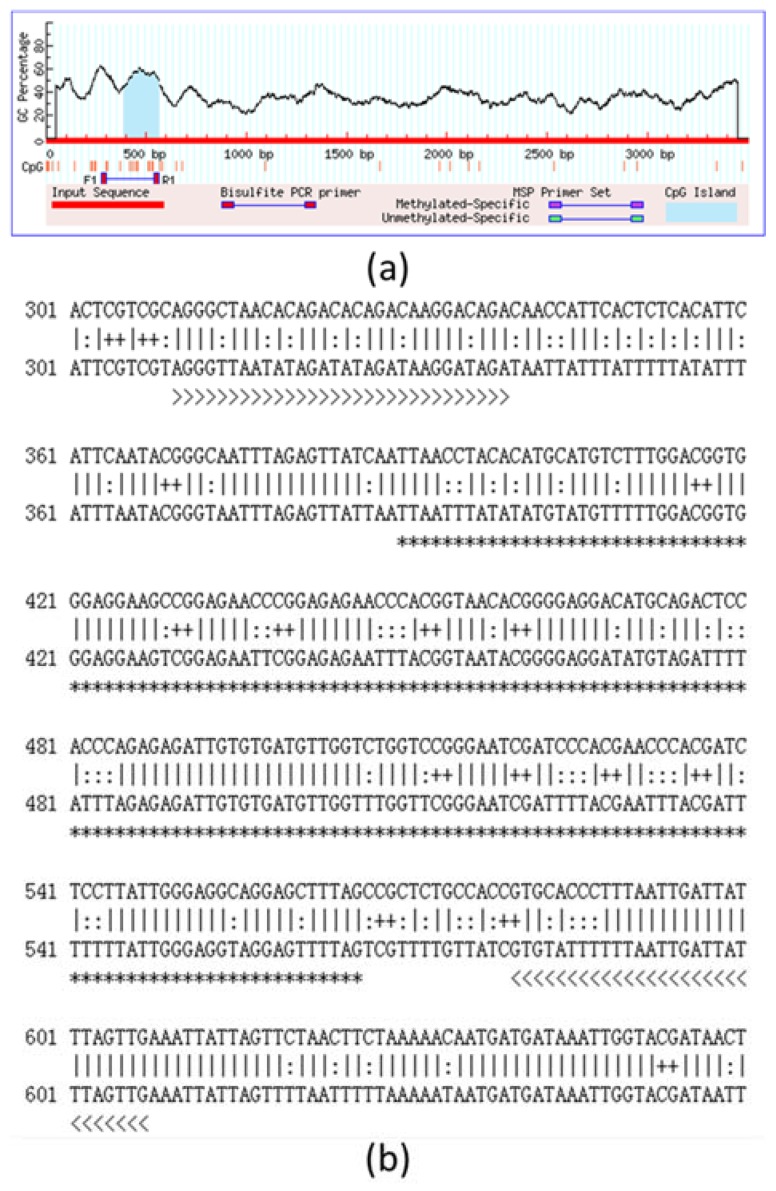
Prediction of the CpG (cytosine–guanine) island, CpG sites, and bisulphite sequencing polymerase chain reaction (BSP) primers. (**a**) The results of online prediction software: the full-length of the CpG island is 177 bp, shown in the blue background region. Nine CpG sites and a pair of BSP primers were obtained. CpG sites are shown by the vertical red short lines ‘**|**’. (**b**) The upper original *T1R1* (taste 1 receptor member 1) sequence was compared with the lower bisulfite modified sequence. For display, assume all CpG sites were methylated and all cytosine was converted into the thymine except for the CpG sites predicted. CpG sites, Non-CpG ‘C’ converted to ‘T’, and the CpG island are represented by symbols ‘++’, ‘:’, and ‘*’, respectively. The given primers, “BSP1 T1R1 F” and “BSP1 T1R1 R”, are represented by symbols ‘>’ and ‘<’, respectively. (For interpretation of the references to color in this figure legend, the reader is referred to the web version of this article.).

**Figure 5:**
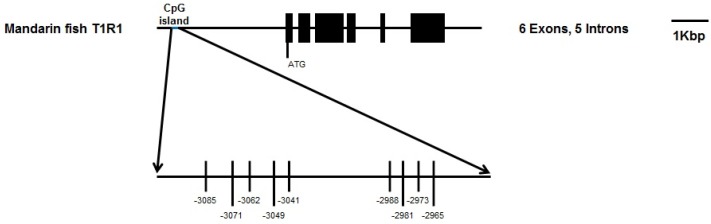
Schematic representation of *T1R1* (taste 1 receptor member 1) gene structure showing the distribution of all nine CpG (cytosine–guanine) sites and the localization of the CpG island. In the present study, initiation codon ATG (translation start site) is regarded as the ‘+1’ site. The CpG island is located in the 5′-flanking region containing nine CpG loci marked by Vertical bars.

**Figure 6:**
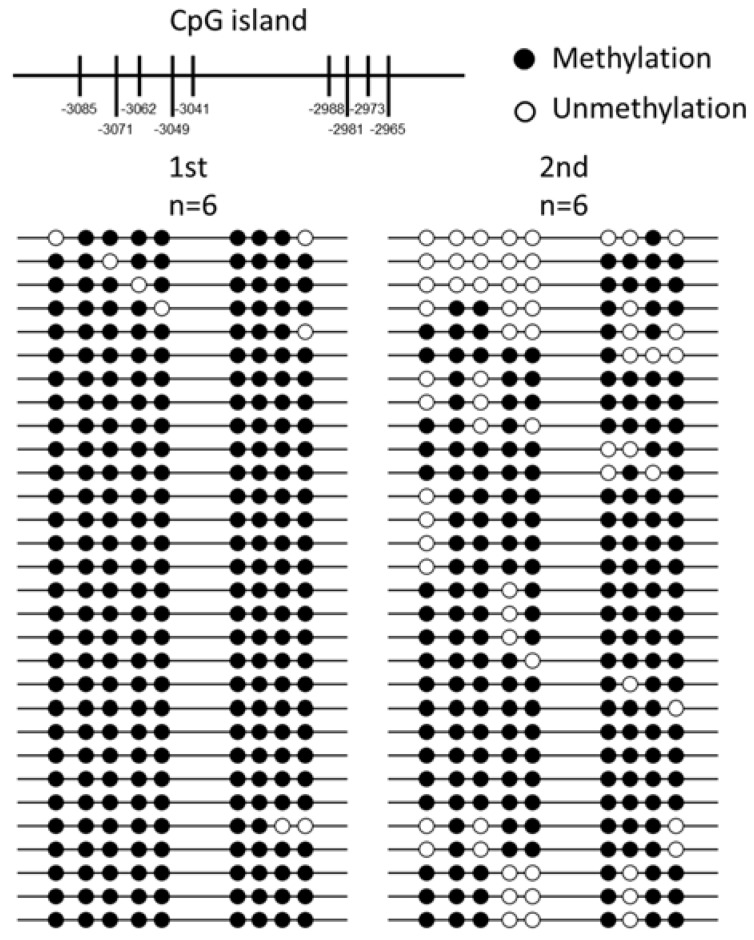
DNA methylation levels at nine candidate CpG (cytosine–guanine) sites contained in the CpG island in the 5′-flanking region of *T1R1* (taste 1 receptor member 1) in the 1st experimental group (trained once) and the 2nd experimental group (trained twice). Each line represents one individual bacterial clone, each circle represents a CpG dinucleotide, white circles represent un-methylated CpG sites, and black circles represent methylated CpG sites.

We analyzed the CpG islands at −3500 bp upstream from the transcription initiation site (designated as 0) of *T1R1* by the methylation analysis software. The prediction of the CpG island, CpG sites, and BSP primers are shown in Figure 4; only one CpG island exists in this region of mandarin *T1R1*. The full-length of the CpG island is 177 bp, the predicted CpG island in the 5′-flanking region from −3191 nt to −2894 nt is shown in Figure 4a, and the length of the product is 298 bp encompassing the whole CpG island (Figure 4b). We analyzed the *T1R1* gene structure and the distribution of all CpG sites, and the localization of the CpG island are shown in Figure 5. The CpG island contained 9 CpG sites: they are located at −3085, −3071, −3062, −3049, −3041, −2988, −2981, −2973, and −2965 nt. Comparison of the DNA methylation levels in brains at the CpG sites in the 1st experimental group (trained once) and the 2nd experimental group (trained twice) is shown in Figure 6. In the 2nd experimental group, the DNA methylation levels in brains at five CpG sites of −3085, −3062, −3049, −3041, and −2981 nt were significantly lower (*P* < 0.05) than those in the 1st experimental group of mandarin fish (Table 1).

## 3. Discussion

To investigate the effect of memory on feeding habit transformation, we compared the success rate of feeding habit transformation to dead prey fish with training of mandarin fish in the 1st experimental group (trained once) and the 2nd experimental group (trained twice). The mandarin fish in the 2nd experimental group had higher success rates of feeding habit transformation from live prey fish to dead prey fish (100%) than those in the 1st experimental group (67%), and shorter reaction time to dead prey fish (<1 s) than those in the 1st experimental group (>1 s). These results indicate that mandarin fish through the first training are much easier and faster to accept dead prey fish during the second training phase. The mandarin fish were able to accept dead prey fish after training, and repeated training promoted the reconsolidation from a labile memory of accepting dead prey fish to a stable memory. Mandarin fish accept live prey fish only in the wild and refuses dead prey fish or artificial diets. Systematic physiological studies have been conducted on the sensory basis of food detection of mandarin fish to elucidate the main reasons for mandarin fish refusing artificial diets [43]. Liang, et al. [3] indicated that mandarin fish yearlings could accept dead prey fish or artificial diets during feeding, using a training program based on its feeding specific sensory modality. Importantly, learning processes (trial-and-error learning and imprinting) play a large role in the behavioral patterns of both fish and mammals [23,24]. The newly learned information is unstable and gradually becomes stable and insensitive to disruption in a the process called memory consolidation [44]. Once memory has stabilized, it might be disrupted but could again become temporally labile if reactivated by recall. Reconsolidation is a process which transforms a reactivated memory from a labile form to a stable one [45,46]. After the first training, the learned information can be stored in the brain of mandarin fish, and the labile memory of accepting dead prey fish can be transformed to a stable form by reconsolidation, the second training. It is suggested that memory might play an important role in the feeding habit transformation from live prey fish to dead prey fish for mandarin fish.

We also examined the mRNA expression of genes involved in synaptic plasticity as well as memory formation in mandarin fish with the feeding habit transformation. The mRNA expressions of *Creb I*, *Bdnf*, and *C/EBPD* genes of mandarin fish in the second training phase (2nd) were significantly increased compared to those in the first training phase (1st). The level of *C/EBPD* gene expression was also significantly increased in the first training phase compared with that of the control group that was not exposed to dead fish. The transcription factor CREB (cAMP responsive element binding protein) is indispensable for long-term memory formation [47], and *Creb* hypomorphic mutant mice (*Mus musculus*) inevitably have impaired spatial and contextual long-term memory formation [48]. *Bdnf* (Brain-derived neurotrophic factor) is one of the target genes of CREB in rat (*Rattus norvegicus*) [49]; the mRNA expression of *Bdnf* gene in the hippocampus is up-regulated in rats after spatial training [50] and contextual conditioning [51]. C/EBPs are expressed especially in neurons and participate in long-term synaptic plasticity of potential memory formation in invertebrates (marine snails *Aplysia californica*) [52,53]. Inhibition of mRNA transcription during training time blocked the long-term memory retention of goldfish (*Carassius auratus*) when using transcriptional inhibitors [54]. Therefore, the enhanced transcription of *Bdnf* and *C/EBPD* genes might be a decisive physiological process for memory consolidation and reconsolidation for accepting dead prey fish and contributing to the feeding habit transformation of mandarin fish.

As the downstream signaling molecular of CREB, the expression level of the *zif268* gene was significantly reduced in mandarin fish during the second training phase (2nd) compared with that of the first training phase (1st), but the mRNA expressions of *Fra2* and *c-fos* were significantly up-regulated. The level of *c-fos* gene expression was also significantly increased in the first training phase compared with that of the control group that was not exposed to dead fish. *Fra2* and *c-fos* belong to immediate early genes as well as the Fos family of transcription factors, and the mRNA expressions of these two genes are increased as a response to various neuronal activation processes, including long term memory [55]. *C-fos* is necessary for consolidation of non-spatial hippocampal-dependent memory [56]. Genetic studies in mice have supported that *zif268* is critical for memory consolidation and long-lasting memory stabilization [57]. *Zif268* knockout mice show impaired long term memories but intact short-term retention [55]. However, the *zif268* mutant mice obtain the ability to learn and form memories after overtraining, suggesting that molecular compensation with learning or memory strategies can recover the loss of the *zif268* gene [55,58]. Interestingly, the level of *zif268* gene expression was significantly up-regulated in the first training phase (1st), but down-regulated in the second training phase (2nd). The basal level of *zif268* gene expression is dramatically and rapidly reduced in the brain of vervet monkeys (*Cercopithecus aethiops*) by systemic administration of N-methyl-D-aspartate receptor antagonists [59] as well as in the brains of rats after monocular deprivation or dark adaptation [60].

Compared with the control group, the gene expression of *SytIV* was significantly increased in the 2nd experimental group after two trainings. SytIV (synaptotagmin-IV) is a membrane trafficking protein; SytIV influences learning and memory by regulating neurotransmitter release and affecting synaptic plasticity [61,62]. Regulation of the BDNF secretion by sytIV is a mechanism that maintains synaptic strength during long-term potentiation in mice [62].

Our results suggest that the increased expression of *Bdnf*, *C/EBPD*, and *Fra2* genes can compensate for *zif268* down-regulation, and the significantly increased expressions of *Bdnf*, *C/EBPD*, and *Fra2* genes of mandarin fish during the second training session might play important roles in memory consolidation of accepting dead prey fish. The behavioral and gene mRNA expression evidence implicating training in learning and memory have revealed a role in the acquisition, consolidation, and subsequent recall of information.

In the present study, compared with the first training phase (1st), the mRNA expression level of the *T1R1* gene was significantly reduced in brains compared to that of the second training phase (2nd) after repeated training. The expression of genes *T1R1*, *T1R2*, and *T1R3* have been detected in different brain regions of mice [35]. The expression of T1Rs in the brain leads to an interesting question about their role. The current nutritional state (such as food deprivation and nutrient excess) regulates expression level of *T1R1* in the mouse hypothalamus [35]. The expressions of T1Rs in the brain might be influenced by animal nutrient sensing. Previous studies [63,64,65] have shown that taste receptors play an important role in the formation of mammalian food habits, and the umami taste receptors T1R1/T1R3 are activated by amino acids, which are preferred tastants. Glutamate binds to nutrient-sensing taste receptor T1R1/T1R3 (L-amino acids) and elicits umami taste, it also regulates the rate of spontaneous firing and functions as a neurotransmitter [66,67]. The brain makes use of nutrient sensing mechanisms that operate in the periphery via taste receptors and downstream signaling molecules [35]. The taste-like signaling mechanisms might be involved in the central regulation in the brain of homeostatic processes. Our results also indicate that the *T1R1* expression in the brain might play important roles in the feeding habit transformation of mandarin fish.

As reported, long-term memory has been found to be involved in various tasks, including social transmission of food preference, object recognition, spatial learning, and conditioned taste aversion [68]. DNA methylation could be involved in the precise regulation of gene expression for adaptation to environmental factors [69]. Previous studies on regulatory mechanisms of DNA methylation have been developed in multiple fields, including ecotoxicology, sexual development, and genetic breeding [70,71,72,73], while scarcely any studies looked at feeding habits and memory formation [74]. The pseudogenization of *T1R1* and nsSNPs in the T1Rs coding region were related to the carnivore-to-herbivore food conversion in giant pandas [38,39] and humans’ ability to taste monosodium glutamate [40,41], respectively. We further found that DNA methylation in the control region of the *T1R1* gene in the brain might be crucial for the reconsolidation and stabilization of the memory of accepting dead prey fish, i.e., feeding habit transformation of mandarin fish. After repeated training, the mRNA expression level of the *T1R1* gene was significantly reduced in the brain, while the methylation levels in the CpG sites of *T1R1* were significantly reduced in brains of mandarin fish. Commonly, cytosine-C5 methylation in the CpG dinucleotides is associated with inhibition of gene expression [75,76,77]. The mechanisms of transcriptional repression by DNA methylation in vertebrates are (1) inhibition of the combination of specific binding factors with their cognate recognition sequences [78] and (2) chromatin remodeling and modification activities repressed by Methyl-CpG-binding proteins and transcriptional co-repressor molecules [79]. Human telomerase catalytic subunit (*hTERT*) expression is a limiting factor in telomerase activity, whereas the *hTERT* promoter is hypermethylated in telomerase-positive tissues and hypomethylated in telomerase-negative tissues, resulting in a contrast with the common relationship between promoter methylation and transcriptional silencing [80]. Partial hypomethylation in the core promoter is essential for *hTERT* expression in spite of methylation preventing binding of the transcriptional repressor CTCF (CCCTC-binding factor) [80]. DNA methylation might play a dual role in some gene transcriptional regulation. The complexity of transcriptional regulation of *T1R1* remains to be explored. There is also a paradox in which promoter-related methylation is inversely correlated with the gene expression, whereas gene-body-related methylation is positively correlated with gene expression [81]. Cytosine methylation in CpG blocks transcription initiation in mammals [82] and transcription elongation in Fungi (*Neurospora crassa*) [83]. The interpretation of the relationship between DNA methylation and gene transcriptional regulation is based on a particular genomic context. The most common breeding strategy for fish is to lay a large number of eggs at one time and then leave the eggs to develop without parental care. The innate patterns of fish maturation might differ from those of mammals learned from their parents [24]. However, the mechanism of DNA methylation on gene expression modulation in fish remains uncertainly understood. Our results suggest that mandarin fish can establish the long-term memory of accepting dead prey fish after repeated training, which might be attributed to gene expression of *T1R1* regulated by DNA methylation in the brain.

In conclusion, mandarin fish through the first training are easier and faster to accept dead prey fish during the second training phase, suggesting that repeated training promotes the reconsolidation from a labile memory of accepting dead prey fish to a stable memory. The expressions of several genes involved in long-term memory formation and feeding habit formation were significantly different in brains of mandarin fish between the first and second trainings. In addition, DNA methylation of the *T1R1* gene might be considered as a regulatory factor for feeding habit transformation from live prey fish to dead prey fish in mandarin fish. These results shed new light on the molecular mechanism of feeding habit transformation in mandarin fish, suggesting the important roles of memory on feeding habit formation.

## 4. Materials and Methods

### 4.1. Fish and Sample Preparation

Experimental mandarin fish (*n* = 30) were obtained from the Wuhan Sihui Fisheries Science and Technology Development Co., Ltd. (Wuhan, China). Mandarin fish were 3 months of age with a total length of 21.22 ± 1.35 cm. Prior to the experiment, each mandarin fish was kept in an independent aquarium (60 × 45 × 45 cm) where it was accommodated to a continuous water filtration system and dissolved oxygen (7.26–7.86 mg/L), temperature (25 ± 1 °C), and pH (7.11–7.59) at constant values. They were fed once daily at 5:30 pm with live India mrigal (*Cirrhinus mrigala*) juveniles as live prey fish for 2 weeks. A total five live prey fish were placed 20 cm away from the mandarin fish. Mandarin fish accepted the live prey fish immediately for feed or had no response, and the live prey fish that had not been eated were left in the aquarium. The uneaten live prey fish were removed from all aquariums at 7:30 pm. Each mandarin fish consumed statistically 2–3 live prey fish every day in the adaptation phase. Samples (*n* = 7) were randomly selected from mandarin fish that was not exposed to dead prey fish as the control group.

The live and frozen India mrigal fry were used as live prey fish and dead prey fish, respectively, in this study, and frozen fry were thoroughly unfrozen before feeding.

All experimental procedures followed the “Guidelines for Experimental Animals” of the Ministry of Science and Technology (Beijing, China) and were approved by the Institutional Animal Care and Use Ethics Committee of Huazhong Agricultural University (Wuhan, China). All efforts were made to minimize suffering. This study did not involve endangered or protected species, thus, no specific permissions were required for the described field studies.

### 4.2. Experiment Test Phase

#### 4.2.1. Pre-Training

There was pre-training before the formal training. Each mandarin fish was kept in an independent aquarium and fed a maximum number of 3 live or dead prey fish once daily at 5:30 pm. A prey fish was placed 20 cm away from each mandarin fish, and only a single live or dead prey fish individual was allowed into each aquarium at any time. Mandarin fish accepted the live or dead prey fish immediately for feed or had no response for 2 min, then, the live or dead prey fish that had not been eaten was removed from the aquarium. The feeding trial was stopped if the mandarin fish had already accepted 3 prey fish for feed. The trail described above was repeated for a maximum of 10 times. The pre-training was performed for 6 days following the accommodative training methods originally described by Liang, et al. [3] as the experimental culture. We strictly followed the procedures of trained steps as follows: (1) day 1, live prey fish fed to satiation only; (2) days 2–4, gradually replacing live prey fish with dead prey fish within the 10 trials day-by-day; (3) days 5–6, only dead prey fish fed. The pre-training phase allowed the mandarin fish to become familiar preying on live or dead prey fish.

#### 4.2.2. Experiment Training Phase 1

The first experiment training (*n* = 23) was performed for 6 days followed the pre-training. Each mandarin fish was kept in an independent aquarium and fed a maximum number of 3 prey fish once daily at 5:30 pm. A dead prey fish was placed 20 cm away from each mandarin fish, and only a single dead prey fish individual was allowed into each aquarium at any time. The feeding habit of mandarin fish was recorded with a digital camera for 2 min. Timing started when the dead prey fish individual was placed into the aquarium. Mandarin fish accepted the dead prey fish immediately for feed or had no response for 2 min. Then, the dead prey fish that had not been eaten was removed from the aquarium. The feeding trial was stopped if the mandarin fish had already accepted 3 dead prey fish for feed. The trial was performed again if the mandarin fish did not eat 3 dead prey fish, and the trail described above was repeated up to 10 times. The reaction time to dead prey fish and the success rate of feeding habit transformation to dead prey fish of mandarin fish were counted from the videos. Regardless of multiple trials, the reaction time for each mandarin fish was recorded as the daily average. As long as at least one dead prey fish was successfully accepted among the trails in the day, the mandarin fish was marked as a successful feeding habit transformer to dead prey fish, whereas the mandarin fish was marked as a failure if it ate no dead prey fish. Samples (*n* = 7) were randomly selected from the remaining mandarin fish for samples of the first training phase as the 1st experimental group (trained once).

#### 4.2.3. Natural Feed Revert Procedure

At the end of the first training phase, the rest of the mandarin fish were returned to the natural feeding habits. Mandarin fish were fed with live prey fish for the next 6 days. Then, in the revert procedure, live prey fish were the first choice of mandarin fish when the live and dead prey fish were presented at the same time, which proves that they had reverted to the characteristics of feeding live prey fish.

#### 4.2.4. Experiment Training Phase 2

After that, the rest of the mandarin fish (*n* = 16) were under the second round of training phase. The procedure of the second training (6 days) was the same as the aforementioned training phase. Samples (*n* = 7) were randomly selected from the remaining mandarin fish for samples of the second training phase as the 2nd experimental group (trained twice).

No mandarin fish died of natural causes during the training phases. The experiment was recorded with a digital camera and the videos were used for subsequent behavior analysis.

### 4.3. Sample Collection

Seven mandarin fish were randomly selected from both the 1st and 2nd experimental groups, respectively. Six samples of mandarin fish were selected for the gene expression levels analysis and DNA methylation analysis. One mandarin fish in each phase was used as a spare sample. At the end of each training phase, the mandarin fish were deeply anesthetized with MS-222 (Argent Chemical Laboratories, Redmond, WA, USA) (200 mg L^−1^) about 2 h after feeding and were killed. The brains of mandarin fish were immediately collected. The mandarin fish brain samples were then frozen in liquid nitrogen and stored at −80 °C for RNA and DNA isolation.

### 4.4. RNA Isolation and Reverse Transcription

Total RNA of mandarin fish brains was extracted with Trizol Reagent (TaKaRa, Tokyo, Japan) according to the manual. The purity and quantity of total RNA were determined using the BioTek Synergy 2 luminometer (BioTek, Winooski, VT, USA), and integrity of total RNA was checked using electrophoresis in 2% agarose gel (Biowest Agarose, Madrid, Spain). The cDNAs were obtained from 1 μg total RNA with the Revert Aid™ Reverse Transcriptase (TaKaRa, Tokyo, Japan) according to the manufacturer’s instructions.

### 4.5. Gene Expression Levels Analysis of Memory Relative Genes in Mandarin Fish

To detect mRNA expressions of memory relative genes in mandarin fish, real-time PCR assays were carried out in a quantitative thermal cycler (MyiQ™ 2 Two-Color Real-Time PCR Detection System, BIO-RAD, Hercules, CA, USA). Primers were designed according to the sequences (Table 2). The *RPL13A* (60S ribosomal protein L13a) gene was used as an endogenous reference to normalize the template amount. All amplifications for each RNA sample were performed in triplicate. Reaction system with 20 μL volume consisted of 10 μL AceQ qPCR SYBR Green Master Mix (Vazyme Biotech Co., Piscataway, NJ, USA), 1 μL of cDNA, 0.4 μL (10 μM) of each primer (Sangon, Shanghai, China) and 8.2 μL of ddH_2_O. The PCR cycling parameters were 95 °C for 3 min, followed by 40 cycles at 95 °C for 10 s, 58 °C for 30 s (according to the annealing temperatures of the different primers), and a melt curve step from 65 °C gradually increasing by 0.5 °C·s^−1^ to 95 °C, with acquisition data every 6 s. Gene expression levels were quantified relative to the expression of the *RPL13A* gene using the optimized comparative Ct (2^−ΔΔCt^) value method. The specificity of the primers was determined through sequencing and melting curve of PCR products. The amplification efficiencies of primers were determined by following the instruction of AceQ qPCR SYBR Green Master Mix (Vazyme Biotech Co., Piscataway, NJ, USA).

### 4.6. DNA Methylation Analysis and Bisulphite Sequencing Polymerase Chain Reaction (BSP)

Six samples of mandarin fish from the first and the second training phases were analyzed, respectively. Genomic DNA was extracted following the standard procedures using TIANamp Genomic DNA Kit (Tiangen, Beijing, China). DNA treatment with sodium bisulphite was performed using the EZ DNA Methylation Kit (Zymo Research, Irvine, CA, USA) according to the manufacturer’s protocol. The sequences of *T1R1* were obtained and submitted to the online software Methprimer (http://www.urogene.org/cgi-bin/methprimer/methprimer.cgi) to acquire the distribution of CpG islands (CGIs) and candidate CpG loci (The parameters are as follows: Island size > 100 bp, GC Percent > 50.0%, Observed/Expected > 0.6). The BSP primers were designed by the online MethPrimer software14 and Primer 5.0; sequences of the PCR primers used for amplifying the targeted products are shown in Table 3. The polymerase chain reaction (PCR) was conducted on Biometra Thermo cyclers (Biometra, Göttingen, Germany) by using Taq plus DNA Polymerase (Vazyme Biotech, Nanjing, China). The PCR protocol was 5 min at 94 °C, 45 cycles of 94 °C for 30 s, 52 °C annealing for 30 s (according to the annealing temperatures of the different primers), and 72 °C for 30 s, with a final extension at 72 °C for 7 min, ending with 16 °C for 10 s. The PCR products were gel purified employing the Gel Purification Kit (Sangon, Shanghai, China) and then were subcloned into the pMD18-T clone vector (Takara, Tokyo, Japan). Five positive clones for each subject were randomly selected for sequencing (Sangon, Shanghai, China). A total of 30 bacterial clones were collected and sequenced in each group. The final sequence results were processed by online QUMA (QUantification tool for Methylation Analysis) software (http://quma.cdb.riken.jp/).

### 4.7. Statistical Analysis

The normality of data was assessed by using SPSS 19.0 software (SPSS, Chicago, IL, USA) with the Shapiro-Wilk test. All data were subjected to one-way analysis of variance using SPSS 19.0 software. Differences between the means were tested by Duncan’s multiple range test (MRT) after homogeneity of variances was checked. The DNA methylation analyses were determined with the χ^2^ test. Statistical significance was determined at the 5% level. All data were presented as mean ± S.E.M (standard error of the mean).

## Figures and Tables

**Table 1:** Methylation status of each CpG (cytosine–guanine) site in the CpG island in the 5′-flanking region of *T1R1* (taste 1 receptor member 1).

CpG Position	−3085	−3071	−3062	−3049	−3041	−2988	−2981	−2973	−2965	Total
Me-CpG 1st ^1^ (%)	29/30	30/30	29/30	29/30	29/30	30/30	30/30	29/30	27/30	262/270
	96.70	100.00	96.70	96.70	96.70	100.00	100.00	96.70	90.00	97.00
Me-CpG 2nd ^2^ (%)	18/30	27/30	22/30	19/30	20/30	27/30	21/30	28/30	24/30	206/270
	60.00	90.00	73.30	63.30	66.70	90.00	70.00	93.30	80.00	76.30
significance	0.007 *	0.236	0.030 *	0.004 *	0.008 *	0.236	0.004 *	1.000	0.470	0.000 *

^1^ The first (fraction) and the second (percentage) lines are for the 1st experimental group (trained once) (*n* = 6); ^2^ The third (fraction) and forth (percentage) lines are for the 2nd experimental group (trained twice) (*n* = 6); * indicates significant differences (*P* < 0.05).

**Table 2:** Nucleotide sequences of the primers for real-time PCR.

Primers Name	Sequences (5′-3′)
RTsc Creb I F	ATACACCCTCCCACTTCA
RTsc Creb I R	TCTCCTCCACATCCGTTC
RTsc Bdnf F	AACTGCCCTCACTCACA
RTsc Bdnf R	ACCTCCCTGGCTCTTAT
RTsc C/EBPD F	GCAGGAGAAGGCGGATTT
RTsc C/EBPD R	CTGGGAAGGCAGGGATGA
RTsc zif268 F	GGATCTTGCCGTGCCTCTTG
RTsc zif268 R	TTGCGACCGCCGTTTCTC
RTsc Fra2 F	CAACCAGGACCTCCAGTG
RTsc Fra2 R	TCTACGCCTTTCAATCTC
RTsc c-fos F	CGATGATGTTTACCGCTTTC
RTsc c-fos R	TAGTATCCCAGATTGTCCC
RTsc SytIV F	TGTCGGAGGATTAGAACG
RTsc SytIV R	CTGAAAGTCCAATGGGTAC
RTsc T1R1 F	TGTATTTTGTTTGATAGAATAAGAGT
RTsc T1R1 R	TAAAAAAACTTAATATAATACTTTTTAAAA
RTsc RPL13A F	TATCCCCCCACCCTATGACA
RTsc RPL13A R	ACGCCCAAGGAGAGCGAACT

**Table 3:** Nucleotide sequences of the primers for BSP (bisulphite sequencing polymerase chain reaction) amplified and DNA methylation analysis.

Primers	Sequences (5′-3′)
*Primers for genomic DNA amplicon*	
CPG1 T1R1 F	AGGGCTAACACAGACACAGACAAGGACAGA
CPG1 T1R1 R	CAACTAAATAATCAATTAAAGGGTGCAC
*Primers for BSP amplicon*	
BSP1 T1R1 F	AGGGTTAATATAGATATAGATAAGGATAGA
BSP1 T1R1 R	CAACTAAATAATCAATTAAAAAATACAC
